# Improvement in osteogenesis, vascularization, and corrosion resistance of titanium with silicon-nitride doped micro-arc oxidation coatings

**DOI:** 10.3389/fbioe.2022.1023032

**Published:** 2022-10-17

**Authors:** Yiding Shen, Kai Fang, Yun Xiang, Keyuan Xu, Liang Yu, Jiaquan Chen, Pingping Ma, Kaiyong Cai, Xinkun Shen, Jinsong Liu

**Affiliations:** ^1^ School and Hospital of Stomatology, Wenzhou Medical University, Wenzhou, China; ^2^ School and Hospital of Stomatology, Zhejiang Chinese Medical University, Hangzhou, China; ^3^ Key Laboratory of Biorheological Science and Technology, Ministry of Education, College of Bioengineering, Chongqing University, Chongqing, China; ^4^ Science and Education Division, The Third Affiliated Hospital of Wenzhou Medical University (Ruian People’s Hospital), Wenzhou, China

**Keywords:** titanium, silicon nitride, corrosion resistance, osteogenesis, angiogenesis

## Abstract

Titanium (Ti) implants have been widely used for the treatment of tooth loss due to their excellent biocompatibility and mechanical properties. However, modifying the biological properties of these implants to increase osteointegration remains a research challenge. Additionally, the continuous release of various metal ions in the oral microenvironment due to fluid corrosion can also lead to implant failure. Therefore, simultaneously improving the bioactivity and corrosion resistance of Ti-based materials is an urgent need. In recent decades, micro-arc oxidation (MAO) has been proposed as a surface modification technology to form a surface protective oxide layer and improve the comprehensive properties of Ti. The present study doped nano silicon nitride (Si_3_N_4_) particles into the Ti surface by MAO treatment to improve its corrosion resistance and provide excellent osteoinduction by enhancing alkaline phosphatase activity and osteogenic-related gene expression. In addition, due to the presence of silicon, the Si_3_N_4_-doped materials showed excellent angiogenesis properties, including the promotion of cell migration and tubule formation, which play essential roles in early recovery after implantation.

## Introduction

With increasing success rates, dental implants have become the treatment of choice to replace missing teeth. Due to its good mechanical properties and biocompatibility, titanium (Ti) has been widely used in dental implants (Chouirfa et al., 2019). However, due to surface bioinertia, osseointegration of titanium implants with surrounding bone tissue after implantation remains challenging (Lei et al., 2021). The lack of angiogenic activity of Ti is also another factor contributing to poor osseointegration, as vascularization plays an important role in assisting bone integration and maintaining bone homeostasis in the early post-implantation period (Zhang and Chen, 2019). Moreover, growing evidence suggests that titanium oxide layers may release ions or particles into adjacent tissues over time, likely caused by corrosion by body fluids (Apaza-Bedoya et al., 2017). All of these can affect implant stability and ultimately cause implant failure.

To solve these problems, many approaches for surface modification have been proposed to improve the physiochemical properties (*e.g.*, roughness, wettability, chemical/crystalline phase composition, *etc.*) of Ti. Ti-based implants with micron/submicron hierarchical structures produced by sandblasting/acid etching (SLA) treatment are representative of commercial implants used in the clinical setting for many years. Since SLA implants are exposed to a body fluid microenvironment rich in chloride ions and proteins after implantation, they also show certain defects caused by corrosion (especially pitting), even those with excellent osteointegration (Zhou et al., 2019; Fang et al., 2021). In addition, the simple preparation process of SLA results in its functional limitations. Thus, further surface modification is required to improve the comprehensive repair potential of Ti implants, especially in cases of complicated microenvironments around the implant (Choi and Park, 2018). In recent years, micro-arc oxidation (MAO) has become one of the best methods to modify Ti-based implants (Li et al., 2004). These Ti-based implants have also been used in the clinical setting. MAO coatings not only have better corrosion resistance but also are beneficial to the adhesion and osteogenic differentiation of osteoblasts due to improved sample surface roughness and energy (Zhang et al., 2004). In addition, by controlling the electrolyte composition, specific oxide layers can be constructed on the Ti surface to provide corresponding properties. Zhang *et al.* designed a Mn-incorporated CaP/TiO_2_ composite coating by MAO, which showed an ideal osteogenic effect (Zhang et al., 2020). Hu *et al.* successfully prepared an MAO porous coating on the Ti-Cu alloy surface to improve the antibacterial activity, mainly due to the formation of Cu_2_O and CuO compounds (Hu et al., 2020).

Recent in-depth studies of inorganic non-metal materials in the field of biomaterials have demonstrated the excellent properties, including osteogenesis and angiogenesis, of silicon and its compounds. Wang *et al.* demonstrated that the incorporation of silicon improved the osteogenic properties of titanium dioxide nanotubes and that the greatest enhancement was observed in the samples with the highest silicon content (Wang et al., 2018). Fu *et al.* reported significantly increased angiogenesis by doping hydroxyapatite-coated Ti implants with silicon (Fu et al., 2020). High-performance ceramic silicon nitride (Si_3_N_4_) has been increasingly favored by researchers for its excellent physiochemical properties, biocompatibility, and osteogenic inductivity (Bal and Rahaman, 2012). Kue *et al.* showed that Si_3_N_4_ significantly promoted human osteoblast-like MG-63 cell proliferation and osteocalcin production (Kue et al., 1999). *In vivo* experiments by Anderson et al. also confirmed the osteogenic ability of Si_3_N_4_ (Anderson and Olsen, 2010). Moreover, as a type of ceramic material, Si_3_N_4_ has attracted research attention for its excellent corrosion resistance. Mazzocchi *et al.* demonstrated the good chemical stability and corrosion resistance of Si_3_N_4_ ceramics as orthopedic implants *in vivo* (Mazzocchi et al., 2008). However, the effects of Si_3_N_4_ on promoting angiogenesis remain unknown. Therefore, we constructed Si_3_N_4_-doped MAO coatings to explore its angiogenesis ability in a material with excellent corrosion resistance and osteoinductive properties.

**FIGURE 1:**
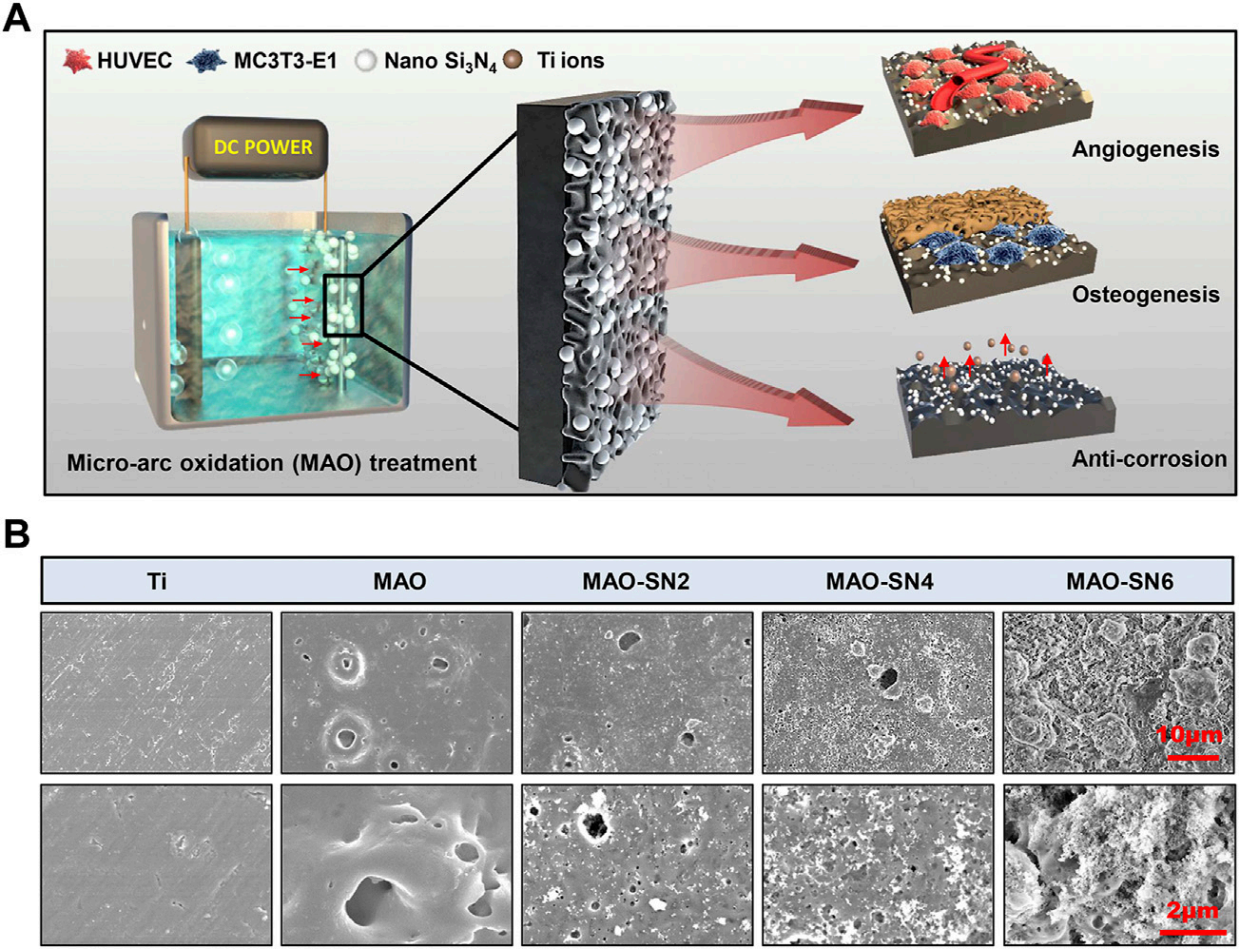
**(A)** Schematic diagram of the micro-arc oxidation treatment of titanium and its osteogenic/angiogenic/corrosion resistant properties. **(B)** Representative SEM images of the Ti, MAO, MAO-SN2, MAO-SN4, and MAO-SN6 substrates.

We successfully constructed a series of Si_3_N_4_ embedded oxide coatings on the Ti surface by MAO (Figure 1A). This study aimed to 1) screen for the optimal Si_3_N_4_ coating and verify its corrosion resistance and 2) further explore the potential of Si_3_N_4_-doped coatings in promoting angiogenesis and osteointegration.

## Materials and methods

### Sample preparation

Commercial pure titanium plates (Northwest Institute for Nonferrous Metal Research, Xi’an, China) with dimensions of 1 cm × 1 cm were used as the substrates for MAO treatment. The plates were polished with sandpaper and washed in acetone, ethanol, and deionized water by ultrasound to remove unnecessary pollutants. The electrolyte solution contained 10 g/L trisodium phosphate (Aladdin Co., China) and deionized water. Different concentrations of Si_3_N_4_ (diameter: 100 nm, cwnano, Shang Hai, China) were added, including 2, 4, and 6 g/L. MAO was applied by DC pulse power for 5 min with a platinum sheet as the cathode. The parameters were an applied voltage of 500 V and fixed duty cycle and frequency of 20% and 1000 Hz, respectively. Depending on the Si_3_N_4_ concentration, the samples were named MAO-SN2, MAO-SN4, and MAO-SN6, respectively. MAO referred to the pristine micro-arc oxidized specimen. After MAO treatment, the samples were cleaned in a 100 W ultrasonic device for 10 min to remove unnecessary impurities or adhered Si_3_N_4_ particles. All samples were sterilized in 75% ethanol and with ultraviolet radiation for 30 min before cell culture.

### Surface characterization

The surface morphology and roughness of samples were observed by scanning electron microscopy (SEM, Zeiss AURIGA FIB, Germany) and atomic force microscope (AFM, Dimension, Bruker, Germany). An instrument to measure contact angle (DSA30, Kruss, Germany) was used to determine the wettability. The chemical compositions of the different coatings were determined by X-ray photoelectron spectroscopy (XPS, Model PHI 5400, Perkin Elmer, United States).

### Si^4+^ release

Phosphate buffered (PBS) solution has a similar ion concentration to physiological liquid and is usually used to model the environment *in vivo*. Therefore, in this experiment, the samples were immersed in PBS solution (1.2 cm^2^/ml) at 37°C. The PBS extract containing Si^4+^ was collected at five different time points (1, 3, 5, 7, and 14 d) and the Si^4+^ concentrations were determined using an inductively coupled plasma optical emission spectrometer (ICP-OES, Avio 500, Perkin Elmer, United States).

### Corrosion test

An electrochemical workstation (LK2005B, Tianjin Lanlike Chemical and Electron High Technology Co., China) was used to test the corrosion resistance. First, a working area measuring 0.5 cm × 1 cm was marked on different substrates, with the remaining uninspected area encapsulated in epoxy resin. Normal saline was used as the electrolyte, a platinum plate as the counter electrode, the sample as the working electrode, and a saturated calomel electrode as the reference electrode. Before measurement, pure nitrogen was filled into the electrolyte for 30 min to reduce the oxygen concentration. After another 30 min to stabilize the current, the Tafel curves of the different samples were scanned at 1 mV/s. Dynamic potential scanning was performed in the potential range of −1.5 V–1.0 V. All operations were performed at ambient temperature. The corrosion potential (Ecorr) and corrosion current density (Icorr) were finally calculated using ImageJ software.

### Osteoinduction ability

#### Cell morphology

The commercial MC3T3-E1 osteoblast cell line (American Type Culture Collection) was cultured at 1 × 10^4^ cells/cm^2^ on the surface of each sample in 24 well plates. After 3 d, the cells were fixed with 4% fixative solution at 4°C for 40 min. Subsequently, 1% Triton X-100 solution was added for 15 min to increase the cell membrane permeability. Tetramethylrhodamine phalloidin (Solarbio Co., China) and 4′,6-diamidino-2-phenylindole (DAPI, Solarbio Co., China) were then added and the samples were incubated in the dark for 40 and 15 min to dye the cytoskeleton and nucleus respectively. Finally, a confocal laser scanning microscope (CLSM, Nikon DS-Ri2, Nikon Instruments Inc., Japan) was used to observe the cell morphology.

#### Cell adhesion

After culturing for 3 h at a density of 2 × 10^4^ cells/cm^2^, the MC3T3-E1 cells were fixed, dyed with DAPI to color the nucleus, and observed by CLSM. The cell numbers were calculated using ImageJ software.

#### Cell viability

Each group of samples was placed into 24-well plates in six replicates. MC3T3-E1 cells (2 × 10^4^ cells/cm^2^) were incubated on each sample to evaluate cell viability. After 3 or 7 d, the media was replaced with 200 μL fresh media and 20 μL 3-(4,5-Dimethylthiazol-2-yl)-2,5-diphenyltetrazolium bromide (MTT) solution. The plates were then incubated for 4 h. The solution was then removed and 1 ml dimethyl sulfoxide (DMSO) was added per well. After incubation on a 37°C shaker for approximately 15 min, 200 μL of the solution was transferred to 96-well plates and read on a microplate reader (Bio-Rad 680, United States) to determine the OD values at 490 nm. All procedures were performed under dark conditions.

#### Alkaline phosphatase activity

After culturing MC3T3-E1 cells (2 × 10^4^ cells/cm^2^) on various samples for 3 and 7 d, an ALP Assay Kit (Nanjing Jiancheng Co., China) was used to determine the level of ALP activity. Briefly, 1% Triton-100 was added to lyse cells for 40 min after washing the samples in PBS. The substrates, deionized water for the blank, standard solution for the control, and corresponding cell lysates for samples were added to 96-well plates, respectively. They were then mixed with buffer solution and Matrix fluid before incubation at 37°C for 15 min. Chromogenic agent was then added and the OD values were measured at 520 nm. The protein content of each sample was also measured using a BCA Protein Assay Kit (Beyotime, China).

#### Mineralization

After culturing MC3T3-E1 cells (2 × 10^4^ cells/cm^2^) for 14 d, the samples were washed with PBS and fixed with 4% fixative solution at 4°C for 15 min. Mineralized nodules on the surfaces were observed by microscope after soaking 20 min in Alizarin Red S solution and thorough washing in deionized water. Finally, cetylpyridinium chloride was added to dissolve the nodule. The OD values were measured at 490 nm.

#### Expression of osteogenic genes

MC3T3-E1 cells (2 × 10^4^ cells/cm^2^) were cultured on the samples for 7 d. Total RNA was extracted using an RNA simple Total RNA kit (Tiangen Biotech Co., China). The total RNA was then reverse-transcribed into cDNA using a PrimeScript RT reagent kit (Takara Bio Inc., Japan). Finally, the expression of target genes [*ALP*, osteocalcin (*OCN*), and osteoprotegerin (*OPG*)] was detected using a SYBR Premix EX Taq Kit (Takara Bio Inc., Japan). The relevant primers are listed in Table 1.

**TABLE 1:** Primer sequences for the MC3T3-E1 cells used in this study.

Target genes	Primers
ALP	F:5′-GAACAGAACTGATGTGGAATACGAA-3′
	R:5′-CAGTGCGGTTCCAGACATAGTG-3′
OCN	F:5′-GAACAGACAAGTCCCACACAGC-3′
	R:5′-TCAGCAGAGTGAGCAGAAAGAT-3′
OPG	F: 5′-GCC​CAG​ACG​AGA​TTG​AGA​G-3′
	R: 5′-CAG​ACT​GTG​GGT​GAC​GGT​T-3′
GAPDH	F:5′-CTCGTCCCGTAGACAAAATGGT-3′
	R:5′-GAGGTCAATGAAGGGGTCGTT-3′

### Angiogenesis induction

#### Preparation of the extract solution

To prepare the extract solution, high-glucose DMEM without fetal bovine serum was applied to the samples at a volume of 1.25 cm^2^/ml. After incubating at 37°C for 72 h, the extract solution was collected.

#### Cell viability

Commercial human umbilical vein endothelial cells (HUVECs, American Type Culture Collection) were cultured at 2 × 10^4^ cells/cm^2^ for 1 d. The media was then to an extraction solution. Cell viability was detected by MTT assay on the third and seventh days.

#### Cell migration

HUVECs (2 × 10^4^ cells/cm^2^) were cultured in 24-well plates in high-glucose DMEM for 3 d until the cells reached 80%–90% confluency. A cross scratch was made using a white spear in every well. The peeling cells caused by the scratch were then gently rinsed with sterile PBS. The cells were cultured in an extraction solution with 1% fetal bovine serum. The wound healing in the same area was observed and photographed using an inverted phase contrast microscope after 0, 12, and 24 h. Finally, ImageJ software was used to measure and calculate the migration areas of the HUVECs.

#### Tube formation

Frozen Matrigel matrix glue was melted in advance at 4°C and added to a 6-well plate (50 μL/well). The plate was then transferred to a 37°C incubator for 30 min. HUVEC cells (1.5 × 10^5^ cells/cm^2^) were inoculated onto the plate with different extract solutions containing 15% fetal bovine serum. Tube formation was observed and photographed using an inverted phase contrast microscope after 0, 3, and 10 h. Finally, Angiogenesis Analyzer in ImageJ was used to measure and calculate the number of nodes (points adjacent to three pixels) and junctions (the border of the overlaying of four nodes), as described previously (Carpentier et al., 2020).

#### Expression of angiogenesis genes

HUVECs (2 × 10^4^ cells/cm^2^) were cultured in an extraction solution containing 10% fetal bovine serum. After 3 d of culture, total RNA was collected and reversed-transcribed to cDNA as described above. Finally, the expression of target genes [vascular endothelial growth factor (*VEGF*), endothelial nitric oxide synthase (*eNOS*), and activin receptor-like kinase 1 (*ACVRL1*)] was detected using a SYBR Premix EX Taq kit (Takara Bio Inc., Japan). The relevant primers are listed in Table 2.

**TABLE 2:** Primer sequence for the HUVECs used in this study.

Target genes	Primers
VEGF	F:5′-AGGGCAGAATCATCACGAAGT-3′
	R:5′-AGGGTCTCGATTGGATGGCA-3′
eNOS	F:5′-GAAGCGAGTGAAGGCGACAA-3′
	R:5′-CCCATTCCCAAATGTGCT-3′
ACVRL1	F:5′-CGCGTGTCACACTTCATGGCTC-3′
	R:5′-ATCAGAAGGCCTTTCCTGGGGG-3′
GAPDH	F:5′-TCAAGAAGGTGGTGAAGCAGG-3′
	R:5′-AGCGTCAAAGGTGGAGGAGTG-3′

#### Statistical analysis

All data were analyzed by one-way analysis of variance (ANOVA) and Student’s t-test, and expressed as means ± standard deviation. *P* < 0.05 was considered statistically significant.

## Results and discussion

### Surface characterization

To detect surface characteristics such as the morphology, roughness, wettability, and chemical composition of the Si_3_N_4_-doped coatings, we performed a series of tests were performed on the samples. As shown in Figure 1B, except for pure Ti, the surfaces of other samples were rough, porous, and volcanic. The surface morphologies were similar to those described by Lou *et al.* (2017), which suggested our successful fabrication of MAO coatings on the surface of Ti. The MAO-SN6, MAO-SN4 and MAO-SN6 groups showed the surface deposition of many nanoscale Si_3_N_4_ particles. The amount of deposition increased significantly with increasing Si_3_N_4_ concentration. The porous MAO surface was almost covered with Si_3_N_4_ nanoparticles in the MAO-SN6 group. MAO is an oxidation process in which the anode metal undergoes arc discharge under high pressure. Because Si_3_N_4_ is negatively charged, it will move to the Ti surface under the action of the electric field and be continuously embedded into the MAO layer in the forms of adsorption, inlay, and molten material wrapping during MAO (Hussein et al., 2013; Shokouhfar and Allahkaram, 2017). The combination Si_3_N_4_ and MAO layer was ensured by a strong mechanical intercalation force (Shokouhfar and Allahkaram, 2016; Xu et al., 2016). Therefore, SEM showed a large amount of Si_3_N_4_ on the surface of the material, ensuring a stable bond between the coatings during implantation. Similarly, Bai *et al.* constructed hydroxyapatite nanorods on the surface of MAO titanium. Their animal experiments showed that the coating integrated well with cells after implantation, significantly promoting osteogenesis (Bai et al., 2018). Shin *et al.* also incorporated ZrO_2_ with MAO, in which ZrO_2_ entered the MAO layer as a mosaic with adsorption similar to that in our study, which enhanced the binding force with the film layer and achieved excellent friction resistance (Shin et al., 2015). In summary, we believed that Si_3_N_4_ had a strong binding force with the MAO substrate based on the mechanical chimerism, with hardness and friction resistance supporting the mechanical behavior of implantation.

**FIGURE 2:**
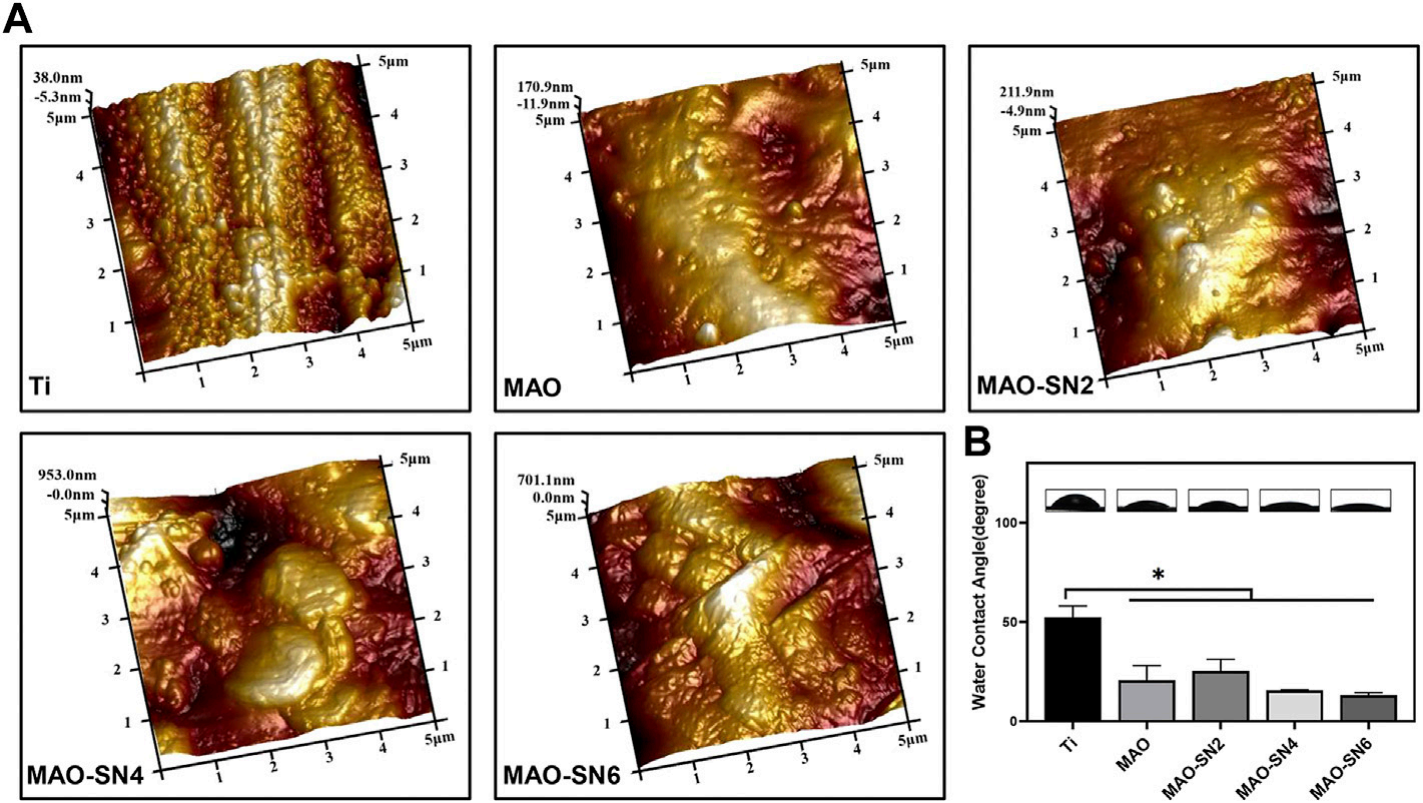
**(A)** AFM images of different samples. **(B)** Representative water contact angle images and quantitative values. *p < 0.05.

Figure 2A shows the surface roughness of different samples. With increased Si_3_N_4_ content, the surface morphology gradually roughened due to the formation of porous surface structures and the deposition of Si_3_N_4_ nanoparticles caused by the MAO treatment. The roughness values were as follows: Ti (Ra 0.033 μm; Rq 0.038 μm), MAO (Ra 0.11 μm; Rq 0.14 μm), MAO-SN2 (Ra 0.13 μm; Rq 0.18 μm), MAO-SN4 (Ra 0.25 μm; Rq 0.33 μm), and MAO-SN6 (Ra 0.28 μm; Rq 0.34 μm).

Assessment of the wettability of the different samples illustrated in Figure 2B showed the highest WCA for pure Ti (∼52.5°). With the continuous doping of Si_3_N_4_, the WCA showed a slightly declining tendency from MAO-SN2 to MAO-SN6. The WCA values were: MAO, ∼20.8°; MAO-SN2, ∼25.6°; MAO-SN4, ∼15.6°; and MAO-SN6, ∼13.2°. After MAO treatment, the hydrophilicity of the materials decreased significantly (*p* < 0.05) compared to pure Ti, but did not differ significantly among the four groups of MAO-treated samples. The possible mechanisms of the hydrophilicity change might be as follows: 1) according to Cassie–Baxter’s solid-liquid-air complex contact model, the droplets were not only in contact with the surface structure but also with the air in the surrounding space (Wenzel, 1936; Cassie and Baxter, 1994) and 2) according to Wenzel’s model and equation, surface hydrophilicity and roughness were positively correlated. Therefore, the emergence of volcanic structures and corresponding rough surfaces after MAO treatment were key to hydrophilic enhancement (Sopata et al., 2021).

**FIGURE 3:**
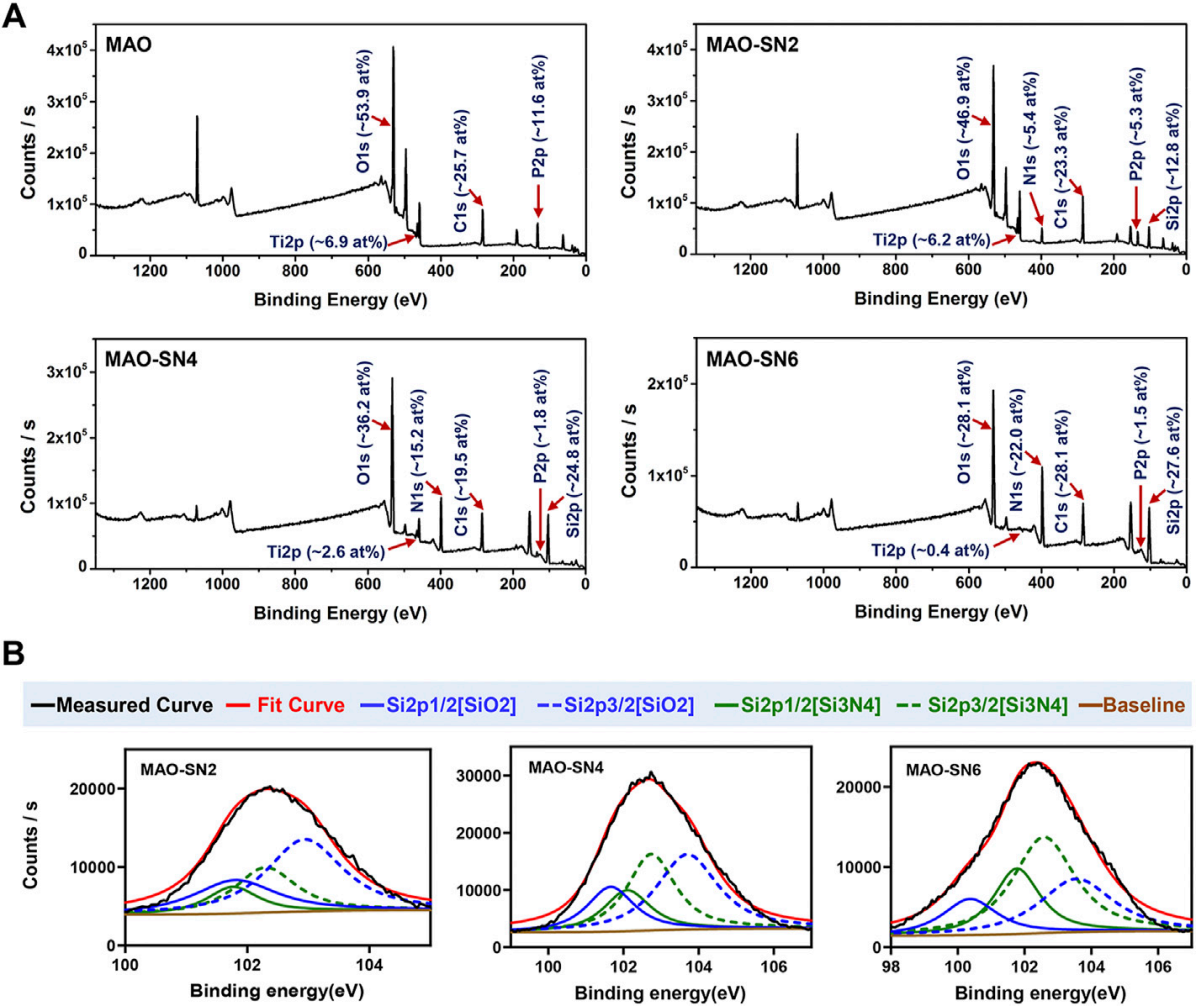
**(A)** XPS patterns and element contents of the MAO, MAO-SN2, MAO-SN4, and MAO-SN6 samples. **(B)** Split-fitting spectra of the Si2p peaks in different groups.

The XPS results of the various MAO-treated coatings are shown in Figure 3 to clarify their chemical compounds. Figure 3A shows the content percentages of each element in the coatings. No elemental Si or N peaks were detected in the MAO group; however, after Si_3_N_4_ doping, Si and N in the MAO-SN2 group reached 12.8 at% and 5.4 at%, respectively. The Si and N content in the MAO-SN4 and MAO-SN6 groups further increased with increasing of Si_3_N_4_ doping concentration [MAO-SN4 (Si: 24.8 at%, N: 15.2 at%), MAO-SN6 (Si: 27.6 at%, N: 22.0 at%)]. In addition, the content of elemental Ti and P in the coating gradually decreased [MAO (Ti: 6.9 at%, P: 11.6 at%), MAO-SN2 (Ti: 6.2 at%, P: 5.3 at%), MAO-SN4 (Ti: 2.6 at%, P: 1.8 at%), MAO-SN6 (Ti: 0.4 at%, P: 1.5 at%)]. Further analysis of Si2p (Figure 3B) showed that the ratio of SiO_2_ to Si_3_N_4_ also gradually decreased [2:1 (MAO-SN2), 1.2:1 (MAO-SN4), 0.6:1(MAO-SN6)].

According to Bock *et al.* (2015), Si-O bonds on the near-surface region of Si_3_N_4_ are chemically equivalent to SiO_2_. Meanwhile, higher temperatures accelerate the oxidization reaction (Tripp and Graham, 1990). MAO treatment often generates electric sparks and releases heat near the anode. Van *et al.* suggested that the local temperature could exceed 2000°C, while Krysmann *et al.* calculated that the temperature reached 8000 K (Krysmann et al., 1984; KurzeKrysmann et al., 1987; Van et al., 1997). Therefore, we believed that SiO_2_ in the target coatings occurred due to the high local temperatures during Si_3_N_4_ oxidation. Moreover, when the Ti sheet was completely covered by the first layer of particles, the surface temperature with which the subsequent Si_3_N_4_ contacted decreased so that less Si_3_N_4_ was oxidized. Therefore, the proportion of SiO_2_ dropped. The reduction of Ti and P elements was caused by the deposition of the Si_3_N_4_ coating, which covered the Ti substrate.

### Cumulative release curve of Si^4+^

**FIGURE 4:**
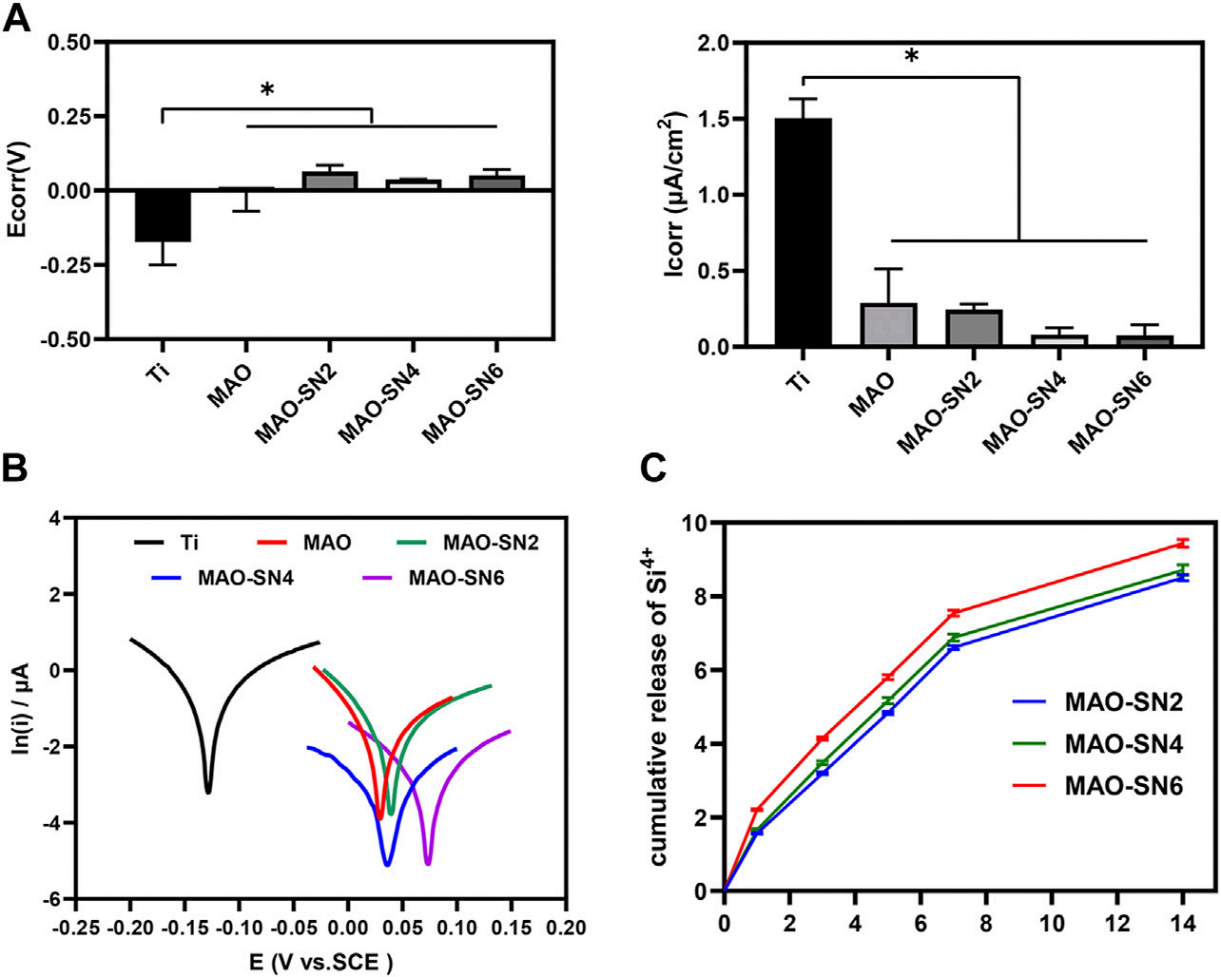
**(A)** Quantitative corrosion current density and corrosion potential. **(B)** Tafel curves of different samples measured in saline solution. **(C)** Release profiles of Si^4+^ from the MAO-SN2, MAO-SN4, and MAO-SN6 samples, *p < 0.05.

Figure 4C shows that Si^4+^ was released rapidly on the first day and continued to be released rapidly for 7 d. The release rate of MAO-SN6 was higher than those of MAO-SN2 and MAO-SN4. After 7 d, the release rate of all groups tended to stabilize and reached the maximum release at 14 d. According to the cumulative release curve, the total Si^4+^ concentrations of MAO-SN2, MAO-SN4, and MAO-SN6 were 8.50 ± 0.03, 8.71 ± 0.06, and 9.44 ± 0.04 ppm, respectively.

### Corrosion test

To investigate the corrosion performance of the different samples, potentiometric scanning in saline was performed. As shown in Figure 4B, the polarization curves of the samples treated with MAO were located further down to the right compared to Ti, which represented a higher voltage and a lower corrosion current. Meanwhile, with the incorporation of Si_3_N_4_, the polarization curve changed more obviously. Further analysis of these data by the Tafel extrapolation method revealed two typical electrochemical parameters of Icorr and Ecorr (Figures 4A). Ti showed the highest Icorr and lowest Ecor values, which differed significantly from those of the other groups (*p* < 0.05). Although the MAO and Si_3_N_4_-doped groups did not differ significantly, the corrosion resistance improved with increasing Si_3_N_4_ concentrations. The Icorr values for Ti, MAO, MAO-SN2, MAO-SN4, and MAO-SN6 were approximately 1.506, 0.290, 0.245, 0.081, and 0.075 μA/cm^2^, respectively. The Ecorr values were approximately -0.174, -0.002, 0.063, 0.038, and 0.052 V, respectively, indicating that the MAO-treated groups had a lower thermodynamic tendency toward electrochemical corrosion (Zeng et al., 2016).

Ti is easily oxidized in air to form a thin surface titanium dioxide passivation layer, which has certain corrosion resistance. However, under complex mechanical stress, the oxide layer is easily destroyed, exposing the pure Ti inside. In the humoral environment, the exposed metal and its metal oxides easily form primary batteries, thereby further accelerating the electrochemical corrosion of implants. Compared to the MAO-treated samples, the TiO_2_ oxide film on the surface of the Ti sample was very thin and unstable in the presence of body fluid; thus, it was the most prone to corrosion among the five materials (Prando et al., 2017). Local corrosion of metal implants occurs through galvanic interactions in the humoral microenvironment (Asri et al., 2017). During MAO, Ti is oxidized to Ti^4+^ and further combined with local anions/O^2-^ to form a uniform and dense ceramic layer, which could significantly inhibit ion diffusion. Lu *et al.* studied the corrosion resistance of pure Ti, MAO, and copper-doped MAO materials under normal saline, hydrogen peroxide, and albumin conditions. Similarly, the corrosion resistance of the materials increased significantly after MAO treatment (Lu et al., 2021). Although MAO specimens already have excellent corrosion resistance compared to pure Ti, micropores and defects inevitably form on their surface due to arc discharge. The micropores and microcracks can become channels for body fluid to enter the membrane and corrode the substrate (Fan et al., 2020). Liang *et al.* also confirmed that chloride ions could enter porous defects on MAO surfaces, which in turn corroded the material (Liang et al., 2010). By incorporating nano-CeO_2_ in MAO, Qin *et al.* effectively reduced the number of cracks and promoted corrosion resistance (Qin et al., 2020). The results of the present study also showed that the continuous doping of Si_3_N_4_ significantly improved the corrosion resistance of the sample. Si_3_N_4_, as a kind of non-oxide ceramic, has good corrosion resistance. As shown in SEM, a large amount of insulating Si_3_N_4_ nanoparticles were embedded into MAO coatings, covering the structural defects and improving the corrosion resistance (Filho et al., 2019). Therefore, the corrosion tendency and corrosion rate of the Si_3_N_4_-doped specimens were significantly reduced, with a Si_3_N_4_ concentration dependence.

### Osteogenesis evaluation *in vitro*

**FIGURE 5:**
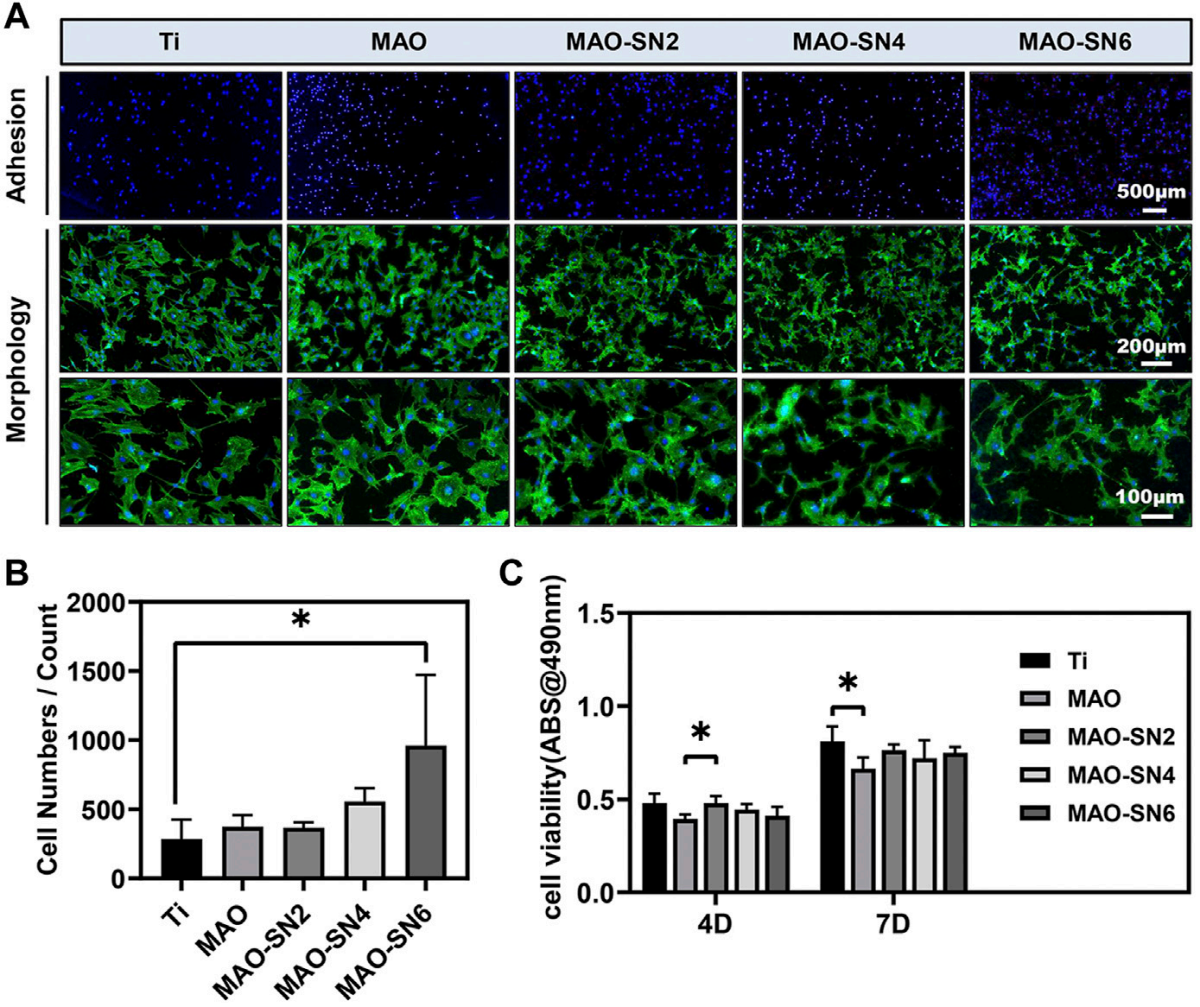
**(A)** Representative staining images of the early adhesion and morphology of MC3T3-E1 cells (green/blue: cytoskeleton/nucleus). Quantitative statistics of cell adhesion numbers **(B)** and cell viability **(C).** *p < 0.05.

To comprehensively evaluate the osteogenic properties of the Si_3_N_4_-modified coatings, we conducted a series of cellular experiments on MC3T3-E1 cells. The CLSM images (Figure 5A) revealed cell adhesion and morphology. The blue and green staining represented the cell nucleus and cytoskeleton, respectively. The density of blue staining increased with increasing Si_3_N_4_ concentration, especially MAO-SN6, suggesting the positive effects of MAO-SN. The images of cell morphology showed the effect of Si_3_N_4_ on cell spread. Overall, we observed no significant change in cell number, while the cell area decreased slightly compared to Ti. Furthermore, we counted the cell numbers to evaluate the effects on cell adhesion. Figure 5B shows that MAO-coated samples doped with Si_3_N_4_ had more cells than pure Ti. The number of cells on MAO-SN6 substrates was the largest and significantly larger than that for Ti (*p* < 0.05). In addition, as shown in Figure 5C, while the cell viability of MAO-treated groups was slightly lower than that of the Ti group, the difference was not statistically significant, similar to the results observed for cell morphology.

**FIGURE 6:**
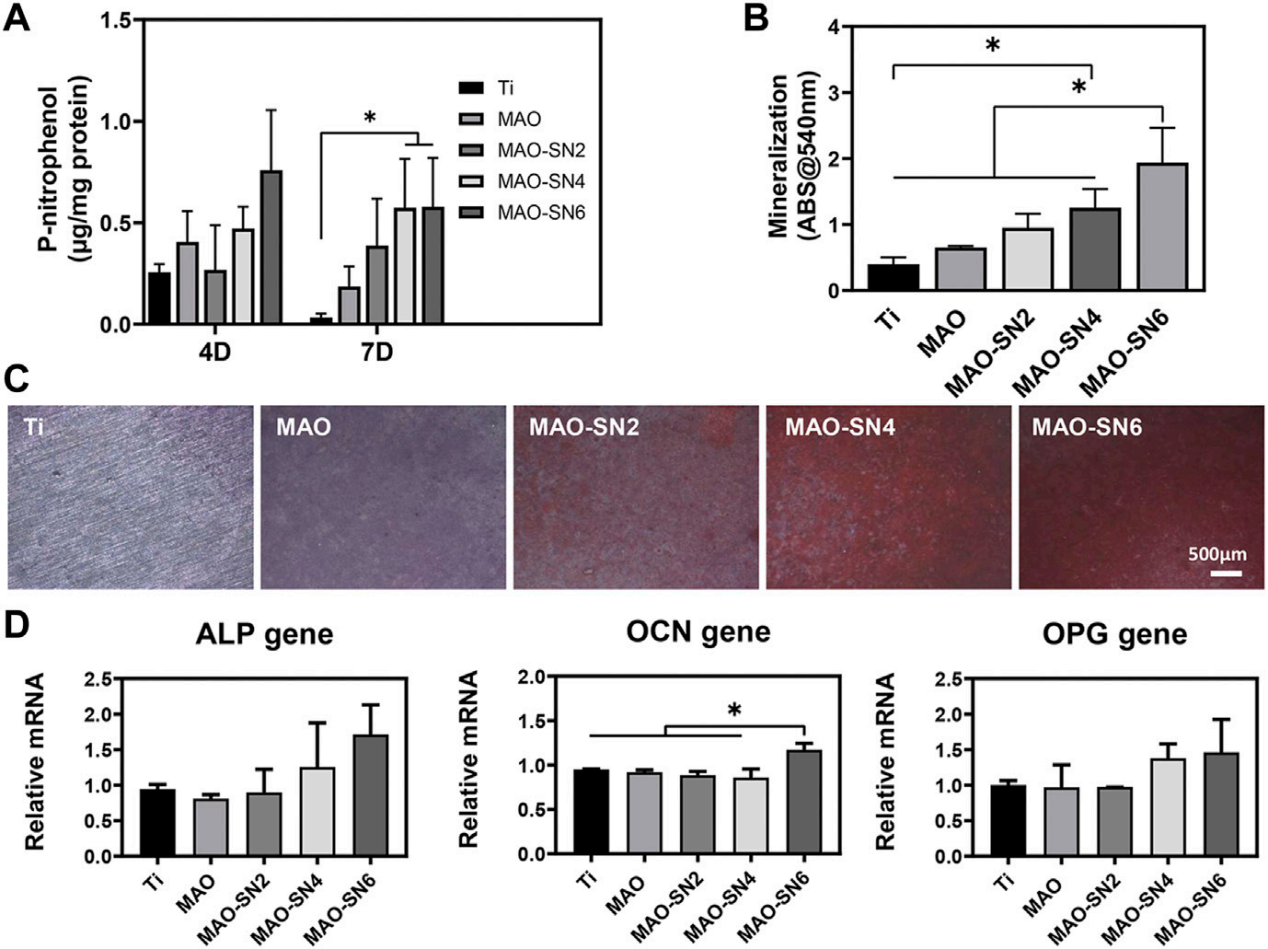
Quantitative statistics of ALP activity at 4 and 7 d **(A)** and mineralization **(B)**. ALP activity at 14 d. **(C)** Representative Alizarin Red S staining images at 14 d. **(D)** Relative expression levels of *ALP*, *OCN*, and *OPG* by MC3T3-E1 cells after 7 d. *p < 0.05.

Next, depending on the ALP and Alizarin Red S results, we further explored the effects in early and late osteogenesis, respectively. As shown in Figure 6A, the P-nitrophenol concentrations showed an overall increasing tendency with increasing Si_3_N_4_ doping. At 7 d, MAO-SN4 and MAO-SN6 showed excellent performance, with significantly better performance for early osteogenesis compared to Ti (*p* < 0.05). Regarding mineralization, the results of the quantitative analysis (Figure 6B) showed the best effects in MAO-SN6 and that both MAO-SN4 and MAO-SN6 significantly ameliorated the mineralization level compared to Ti (*p* < 0.05). The light microscopy images (Figure 6C) further showed similar trends. In addition, as shown in Figure 6D, compared to Ti, MAO-SN6 increased the expression of osteogenesis-related genes, including *ALP*, *OCN*, and *OPG*.

In conclusion, the incorporation of Si_3_N_4_ in MAO coating resulted in significantly improved cell adhesion. Dai *et al.* coated Si_3_N_4_ on PEEK surfaces through suspension coating and melt binding to promote MC3T3-E1 adhesion and proliferation (Dai et al., 2019), consistent with our results. Regarding the mechanism of this finding, previous studies showed that the adsorption of proteins on the surface of biomaterials promoted contact between the cell membranes and biomaterials, thereby enhancing cell adhesion. Si_3_N_4_ has a good affinity for albumin and other proteins (Formentin et al., 2018; Sainz et al., 2020), which might promote the absorption of proteins on the Si_3_N_4_-modified coatings, thus promoting cell adhesion. In addition, as shown in Figures 2B,C, the MAO treatment significantly enhanced the surface roughness and hydrophilicity, which might also promote cell adhesion/proliferation. Increased surface roughness reportedly promoted osteoblast spread and migration (Jiang et al., 2013; Abar et al., 2021). In the cell viability tests, as an inorganic nanomaterial with excellent biocompatibility, Si_3_N_4_ did not show significant cytotoxicity, consistent with previous reports (Bock et al., 2015; Fiani et al., 2021; Lee et al., 2021). Regarding osteogenic differentiation, Si_3_N_4_ doping significantly promoted ALP and mineralization compared to the control group, possibly due to the slight dissolution of Si_3_N_4_ in the body fluid environment and the slow release of Si^4+^. Previous studies reported that certain concentrations of silicon ions released by silicon-based biomaterials could effectively enhance cell proliferation and differentiation (Shie et al., 2012; Wu et al., 2019). In addition, *ALP*, *OCN*, and *OPG* expression levels increased significantly and showed a certain dose dependency (except for *OCN*). Similar results were reported by Xu *et al.* (Xu et al., 2019). In summary, Si_3_N_4_ modification not only promoted cell adhesion/proliferation but also enhanced osteogenic differentiation by increasing *ALP*, *OPG*, and *OCN* expression.

### Vascularization evaluation

**FIGURE 7:**
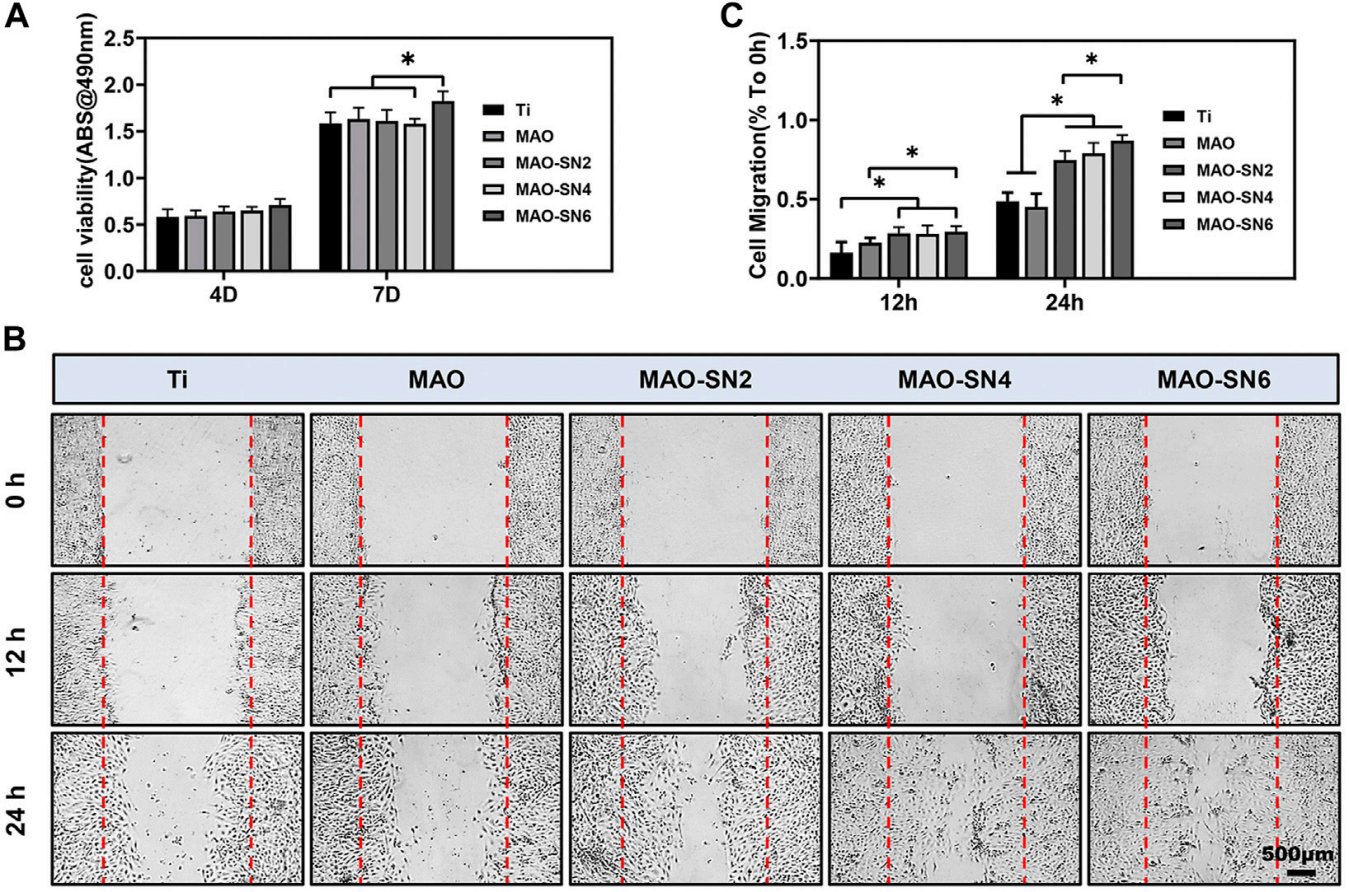
**(A)** Viability of HUVECs in different extracts after 4 and 7 d. **(B)** HUVEC migration after 0, 12, and 24 h. **(C)** Quantitative analysis of migration areas after incubation in different extracts for 12 and 24 h. *p < 0.05.

To fully consider the vascularization ability of Si_3_N_4_-added coatings, we conducted a series of cellular experiments on HUVECs. Micro vessels beside damaged tissues are mainly regenerated by sprouting; that is, the endothelial cells of existing blood vessels are activated to proliferate and migrate to form new blood vessels (Gurevich et al., 2018). Because endothelial cells do not contact the surface of titanium, we performed cell experiments with extract solutions. Cell viability (Figure 7A) did not differ notably among all groups at 4 d. However, MAO-SN6 was significantly superior to Ti and MAO-SN4 (*p* < 0.05) after 7 d, indicating the good effects of MAO-SN6 on cell viability in HUVECs.

Next, cell migration experiments were used to explore wound healing. As shown in Figure 7C, after incubation for 12 h, no obvious cell migration was observed in any group. The MAO-treated groups showed slightly better results than the Ti group. After incubation for 24 h, the cell migration abilities of the Si_3_N_4_ doping groups were significantly better than those of the Ti and MAO groups. The scratch wounds in the MAO-SN6 group basically healed, showing the best effect, followed by the MAO-SN4 group. Figure 7B further shows the percentages of HUVEC migration areas in each group compared to those at 0 h. In the first 12 h, the migration rate of the cells in each group was low. However, within 12 h–24 h, the cell migration rate accelerated. The percentages of migration area in the MAO-SN2, MAO-SN4, and MAO-SN6 groups were 1.5, 1.6, and 1.7 times that of the Ti group and 1.8, 1.9, and 2.1 times that of the MAO group, respectively.

**FIGURE 8:**
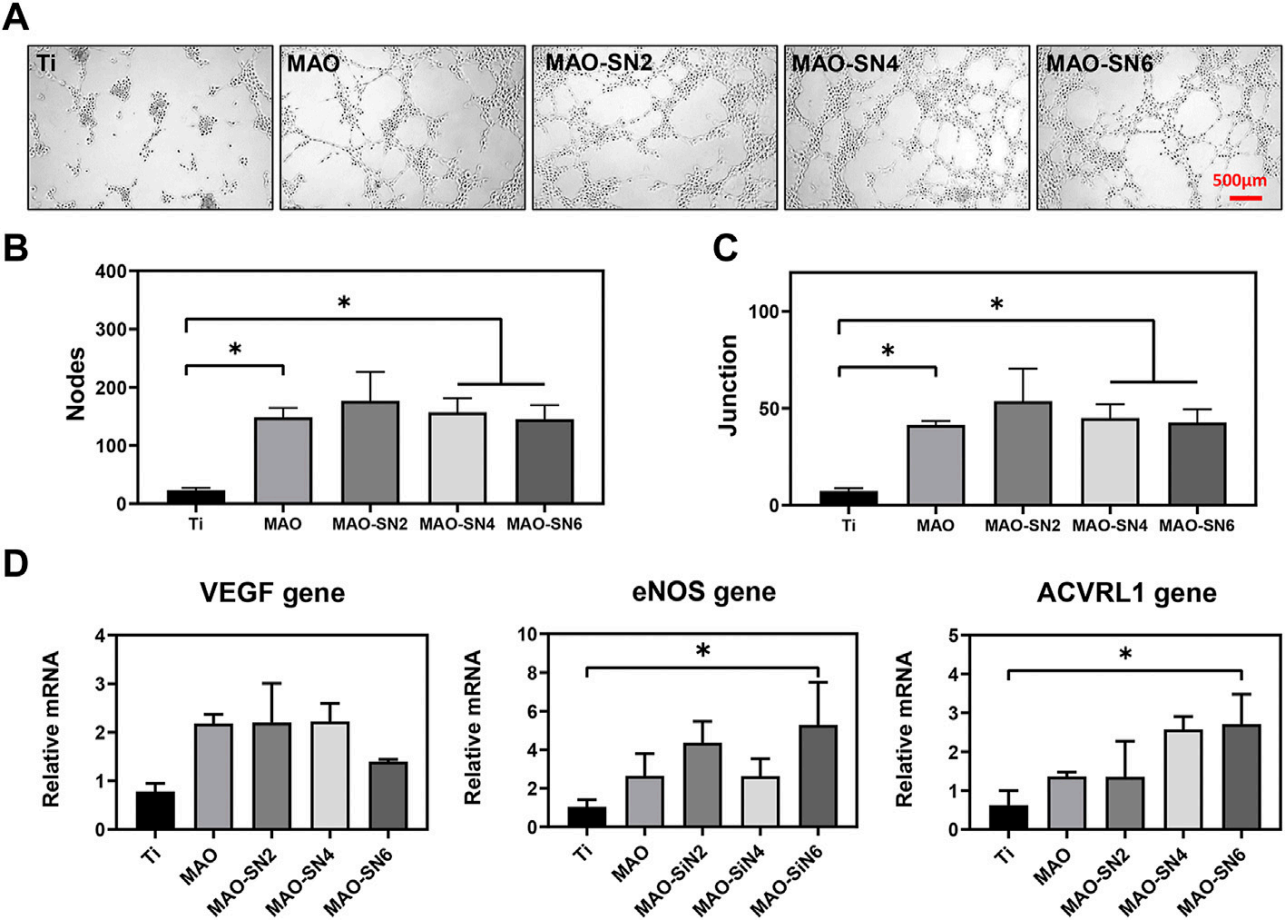
**(A)** Tube formation after 6 h. Quantitative analysis of tube formation according to node **(B)** and junction **(C)** numbers. **(D)** Relative expression levels of *VEGF*, *eNOS*, and *ACVRL1* by HUVECs after 3 d. *p < 0.05.

In addition, the tubule formation experiment is a rapid method to measure angiogenesis ability *in vitro*. HUVECs are connected into lines, in which the nodes represent typical early-stage angiogenesis. In the middle and late stages, vascular branches are cross-linked to form a vascular network structure (Sriphutkiat et al., 2019). As shown in Figure 8A, the Ti group had fewer junction points and tubules, while the other four MAO-treated groups had significantly more related structures. The cells were closely linked to form a complex network of blood vessels. The quantitative results of the junction points and branches in each group are shown in Figures 8B,C. Moreover, while the numbers of nodes and connection points formed in the extract of the four MAO-treated groups were significantly higher than those of the Ti group, no significant differences were observed among them. The results of PCR to further verify and analyze the effect of samples on angiogenesis at the molecule level are shown in Figure 8D. VEGF is considered the most important angiogenesis regulator (Zhou et al., 2018). *eNOS* activation and expression were mainly based on PI3K/Akt/eNOS-dependent signaling and played an important role in the first 72 h before blood vessel formation. *eNOS* also mediated NO synthesis, which is essential for endothelial cell proliferation and migration (Yang et al., 2018). *ACVRL1* is related to hereditary hemorrhagic telangiectasia. Si_3_N_4_ promoted blood vessel dilation. With increasing Si_3_N_4_ concentration, gene expression gradually increased (García-Sanmartín et al., 2022). Figure 8D shows that compared to Ti, the four MAO-treated groups (MAO, MAO-SN2, MAO-SN4, and MAO-SN6) showed higher levels of gene expression to different degrees, including *VEGF*, *eNOS*, and *ACVRL1*. MAO-SN6 showed the best effect, with significance in *eNOS* and *ACVRL1* but not *VEGF*.

The above results suggested that Si_3_N_4_-doped MAO treatment had a strong ability to promote angiogenesis, including earlier migration and tubular formation of endothelial cells. This might be due to Si^4+^ arising from a slight melting of Si_3_N_4_, as confirmed by the release results (Figure 4C). Relevant studies have shown that Si^4+^ can promote angiogenesis. For example, Rubio *et al.* reported the synthesis of SiO_2_-chitosan coatings on Ti implants, which showed effective Si^4+^ release from the coatings to promote bone formation (Palla-Rubio et al., 2019). He *et al.* described the formation of a silicon-doped micro nano-structure on a Ti implant, in which the nanostructure above the microporous protected the rapid release of Si^4+^ to achieve the long-term sustained release. Thus, it further promoted cell proliferation and differentiation (He et al., 2018). Moreover, although the MAO group showed no significant effect on HUVEC migration, it showed a certain positive effect on tubular formation. This may occur due to the formation of a small amount of TiO_2_ during anodic oxidation. TiO_2_ particles have also been confirmed to trigger the generation of reactive oxygen species (ROS) (Grande and Tucci, 2016). Osumi *et al.* reported that although excessive ROS could lead to cellular damage, physiological levels of ROS mediated beneficial cellular responses, including angiogenesis (Osumi et al., 2017).

## Conclusion

In this study, we designed a series of Si_3_N_4_-doped coatings on Ti by MAO. Si_3_N_4_ oxidation at local high temperatures finally formed a mixture of Si_3_N_4_ and SiO_2_. The Si_3_N_4_-doped samples showed better hydrophilicity compared to pure Ti due to improved surface roughness and promoted cell adhesion without damaging cell viability. The results of the osteogenesis experiments indicated that the Si_3_N_4_ modification performed well in the early and late stages of osteogenic differentiation in a dose-dependent manner. These experiments also showed good angiogenesis ability, including cell migration and tubular formation, by releasing silicon ions. The results of the corrosion test verified excellent corrosion resistance of the Si_3_N_4_-doped coatings. Therefore, the multifunctional Ti-based implant showed good osteogenesis, angiogenesis, and corrosion resistance, which suggested its potential in clinical applications.

## Data Availability

The original contributions presented in the study are included in the article/Supplementary material. further inquiries can be directed to the corresponding authors.
